# Gender Influences on Brain Responses to Errors and Post-Error Adjustments

**DOI:** 10.1038/srep24435

**Published:** 2016-04-14

**Authors:** Adrian G. Fischer, Claudia Danielmeier, Arno Villringer, Tilmann A. Klein, Markus Ullsperger

**Affiliations:** 1 Otto-von-Guericke University, Institute of Psychology, D-39106 Magdeburg, Germany; 2 Center for Behavioral Brain Sciences, D-39106 Magdeburg, Germany; 3 University of Nottingham, School of Psychology, Nottingham NG7 2RD, UK; 4 Radboud University, Donders Institute for Brain, Cognition and Behaviour, 6525 HR Nijmegen, The Netherlands; 5 Max Planck Institute for Human Cognitive and Brain Sciences, Department of Neurology, D-04103 Leipzig, Germany; 6 Day Clinic for Cognitive Neurology, University Clinic Leipzig, D-04103 Leipzig, Germany

## Abstract

Sexual dimorphisms have been observed in many species, including humans, and extend to the prevalence and presentation of important mental disorders associated with performance monitoring malfunctions. However, precisely which underlying differences between genders contribute to the alterations observed in psychiatric diseases is unknown. Here, we compare behavioural and neural correlates of cognitive control functions in 438 female and 436 male participants performing a flanker task while EEG was recorded. We found that males showed stronger performance-monitoring-related EEG amplitude modulations which were employed to predict subjects’ genders with ~72% accuracy. Females showed more post-error slowing, but both samples did not differ in regard to response-conflict processing and coupling between the error-related negativity (ERN) and consecutive behavioural slowing. Furthermore, we found that the ERN predicted consecutive behavioural slowing within subjects, whereas its overall amplitude did not correlate with post-error slowing across participants. These findings elucidate specific gender differences in essential neurocognitive functions with implications for clinical studies. They highlight that within- and between-subject associations for brain potentials cannot be interpreted in the same way. Specifically, despite higher general amplitudes in males, it appears that the dynamics of coupling between ERN and post-error slowing between men and women is comparable.

Sex differences on brain function^1^, structure^2^,^3^, and its genetic associations^4^ as well as differential gender effects in various psychiatric diseases are inexorably moving centre stage^5^. Among these diseases of high scientific and societal relevance and for which sex effects in prevalence, prognosis, and treatment responses are known, are ADHD^6^, substance abuse^7^, schizophrenia^8^ and depression^9^. Furthermore, alterations in performance monitoring functions, an essential feature that provides the means to quickly react to unintended action consequences^10^, are being investigated in all of these disorders^11^,^12^,^13^,^14^,^15^,^16^. However, gender differences in core performance monitoring functions, which may help to map symptomatology to physiologic processes, are poorly understood, and – despite promising early results – have rarely been tested in large samples. Such findings are especially important in the National Institute of Mental Health’s framework of Research Domain Criteria (RDoC)^17^, which attempts to understand neurobiological correlates of psychiatric symptoms. Furthermore, there is considerable interest in understanding behavioural and neurophysiologic differences between men and women, and the existence of dimorphic brain features is currently a matter of extensive research interest and debate^2^,^3^.

Detection of errors and subsequent behavioural adjustment is a cognitive process with well established neural correlates regarding their localization^18^ and precise time courses^19^. Human electrophysiological studies established the error-related negativity (ERN, peaking between 50 and 100 ms after the erroneous response) and a consecutive error positivity (Pe, 100–250 ms after error) as valid markers of objective and accumulated subjective evidence of action errors, respectively, and predictors of consecutive behavioural adjustments^20^,^21^. Such adjustments are reflected in increased reaction times (RTs) after errors, known as post-error slowing (PES), which is thought to represent flexible, unspecific adjustments^15^,^22^. Some studies found that PES is associated with increased performance accuracy following errors (PIA), which would render PES an adaptive strategy^23^. Despite valid paradigms and concepts, studies of sex differences with regard to these processes have so far yielded inconclusive results. For example, one study found that women display increased post-error slowing following failed inhibitions in a stop-signal task^24^, while two other studies found no such difference employing either also a stop-signal task^25^ or a flanker task^26^. The latter study also found increased ERN as well as Pe amplitudes for male participants^27^, while another study reported the opposite finding^28^. Furthermore, some studies suggest generally longer RTs in female subjects^29^, yet others attributed this finding to decreased distractibility in males by task irrelevant cues^30^,^31^ (but see^32^). This reduced distractability has been interpreted as evidence for the ‘extreme male brain’ hypothesis, which states that autism reflects the extreme of the normal male profile^33^, thus linking gender differences and neurological disorders. Because of these diverging findings on both neural and behavioural aspects, which are likely intertwined, we used a different analysis approach, namely multiple regression analyses, to rule out influences of confounding factors for behavioural and ERP analyses and to disentangle possible explanations for gender differences.

Here, we present data from an investigation of gender differences in performance monitoring including high-density electroencephalographic (EEG) recording. A speeded arrow-version of an Eriksen Flanker Task was performed while EEG was recorded from 874 participants (438 female, 436 male) constituting by far the largest sample investigating sex effects on performance monitoring and its neural correlates to date. Generally, errors in this task are induced by presenting distracting flanking stimuli in visual proximity and slightly ahead in time of the imperative, central target stimulus, thereby inducing conflicting response tendencies. We employed a manipulation of stimulus distances among each other (close and far) and response stimulus intervals (short and long) in order to modulate the amount of response conflict and optimize measurable PES^34^. Furthermore, in order to disentangle neural processing of action errors from confounds, such as RTs, we controlled for these factors using multiple regression techniques on the EEG data both within and across subjects.

## Results

For behaviour data analysis, we compared mean RTs for correct responses between male and female subjects as well as error rates and post-error slowing (calculated as the difference in RT between post-error and post-correct trials). Therefore, three regression analyses as well as a control analysis (see Methods) were calculated and we report Bonferroni corrected p-values for 14 tests as well as 99.9% confidence intervals (CI). All of these analyses included age as a separate factor as well as other regressors to increase the specificity of the observed effects (see below). Results of overall task performance are reported in the Methods and Supplemental Material sections.

### Error Rate and RT

**Figure 1:**
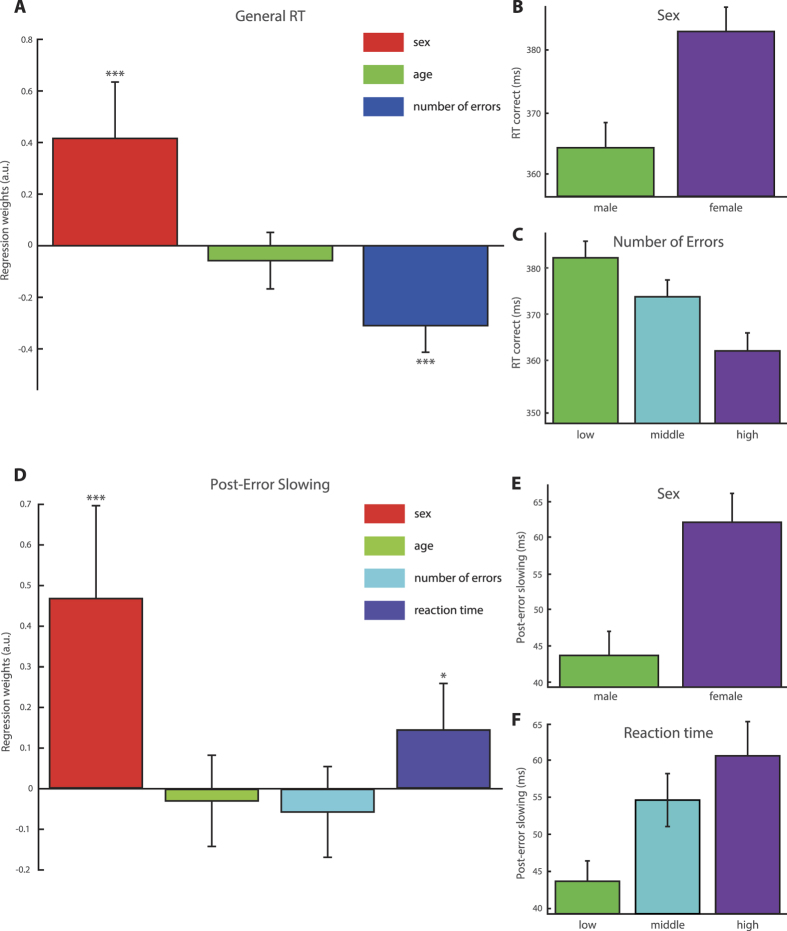
Sex Effects on Reaction Time and Post-Error Slowing. (**A**) shows the regression weights of factors sex, age, number of errors on correct trials’ RT revealing a significant effect for factor sex using multiple linear regression analysis. (**B**) RT broken down by factor sex showing that male subjects responded on average ~16 ms faster than female subjects. (**A**,**C**) Participants who committed fewer mistakes also responded slower indicating a speed accuracy trade-off. There was no effect of age on RT (**A**). (**D**) Female participants displayed significantly higher post-error slowing, which corresponds to an increase in RT of 20 ms compared to male subjects (**E**). The general RT also had a small effect on post-error slowing and participants who responded slower displayed higher post-error slowing (**F**). Note that for the analysis presented in (**A**) all correct trials following errors were excluded and thus the higher RT seen in female subjects cannot be explained by the post-error slowing effect. (**A**,**D**) display regression weights while (**B**,**C**,**E**,**F**) display raw values. Error bars = 99.9% CI, * = p < 0.05, ** = p < 10^−4^, *** = p < 10^−8^ following Bonferroni correction.

First, we found that the total number of errors committed in the task was not modulated by the factor sex (b = 0.04, p = 1, 99.9% CI = −0.19–0.27) and males committed on average 155 (14.4%) and females 153 (14.2%) errors. For general RT, we included gender, age and the number of errors into a linear regression model. This revealed that male subjects responded on average around 16 ms faster on correct trials compared to female subjects (Fig. 1, b = 0.42, CI = 0.20–0.64, p = 7.39 × 10^−9^). Participants’ age had no effect on RT (b = −0.06, CI = −0.17–0.05, p = 1). Subjects who made fewer errors also responded slower on correct trials (b = −0.31, CI = −0.41– −0.21, p = 9.63 × 10^−21^) indicative of individual differences in emphasizing speed or accuracy. Note that we excluded all post-error trials from this analysis to not confound the results with error-induced RT changes. On errors themselves, male subjects again responded faster (ΔRT = 9 ms, b = 0.24, CI = 0.01–0.46, p = 0.009).

### Post-Error Slowing

For post-error slowing, we included age, the number of errors, as well as the mean RT into the model in order to investigate whether sex had an effect on post-error slowing over and above the observed difference in RT. We found that the amount to which female subjects slowed their responses down following mistakes was significantly larger compared to male subjects (b = 0.47, CI = 0.24–0.70, p = 4.29 × 10^−10^). This corresponded to a post-error slowing increase of 20 ms or 42% compared to male subjects (Fig. 1D,E). Neither age (b = 0.03, CI = −0.15–0.08, p = 1) nor the number of errors committed (b = −0.06, CI = −0.14–0.08, p = 1) had an effect on post-error slowing. However, subjects with generally higher RT also showed higher post-error slowing (b = 0.14, CI = 0.03–0.26, p = 0.0006). We furthermore conducted a control analysis by calculating post-error slowing with respect to the error preceding trial^35^, which accounts for possible general shifts in attention during the task. However, this did not qualitatively alter results (see Methods and Supplemental Material). We also normalized PES by each subjects’ RT on correct trials by dividing the PES measure by the mean standard trials’ RT^36^ to account for differences in general RTs. Again, results remained qualitatively unchanged demonstrating a larger RT increase in women (16.1 ± 1.0%) compared to men (11.9% ± 0.9%, b = 0.47, CI = 0.23–0.70, p = 1.05 × 10^−9^). Furthermore, an exploratory analysis of sex effects in post-error differences in accuracy revealed no sex effect on post-error increases in accuracy (PIA; corrected p = 1).

### Analysis of Distractibility

As it has been reported that women are more distracted by irrelevant and conflicting task information, we compared RT increases induced by the congruence of the presented stimuli. Incongruent trials led to higher RT across subjects (ΔRT = +62 ms, *t*_873_ = 121.5, p = 0 within machine precision) and we analysed the difference between congruent and incongruent trials (congruency effect). There was a small but significant sex effect (b = 0.30, CI = 0.06–0.54, p = 0.0004), which was caused by women displaying on average a 5 ms larger congruency effect. Furthermore, we tested whether the overall gender-related RT difference was found on both congruent and incongruent trials, and the gender effect remained significant in both cases (ps < 10^−5^).

### Error Related Brain Activity

**Figure 2:**
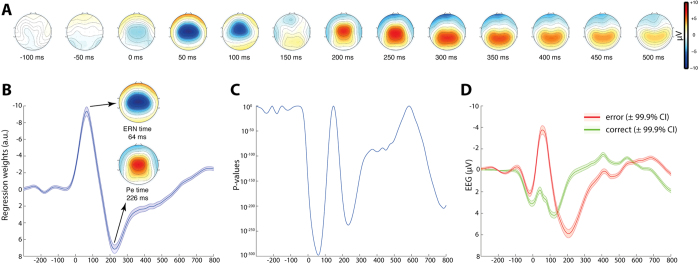
Error Effects on EEG Activity. Scalp topographies of response-locked regression weights show the classical ERN and Pe succession (**A**). Maxima for ERN and Pe were found at 64 ms and 226 ms, respectively, which both displayed central scalp topographies (**B**). Associated p-values for t-tests of within subject regression weights for a difference from zero are displayed with logarithmic scaling in (**C**). (**D**) Shows regular ERPs, which do not account for error-unspecific task effects (see Supplemental Material for details).

We used a two-stage analysis approach: first we identified time and location (i.e. electrodes) of maximum error-related activity in the task and then used these for analysis of second level effects. Therefore, we employed single-trial robust regression to obtain a regression weight time-course for error-related activity locked to response onset for all electrodes^37^. This model included various regressors to control for possible confounds such as each trial’s congruency, flanker distance, and reaction time (see Methods and Supplemental Material for more information about the model). First level regression weights were scaled by their respective standard errors and thus are comparable across subjects and regressors. From this model (Fig. 2A,B) we found the maximum amplitude of negative-going error-related EEG activity at electrode Cz 64 ms following response onset – compatible with the ERN. This was followed by a consecutive positive covariation peaking at 226 ms again at electrode Cz, reflecting the Pe.

### Gender Differences in Error-Related Brain Activity

**Figure 3:**
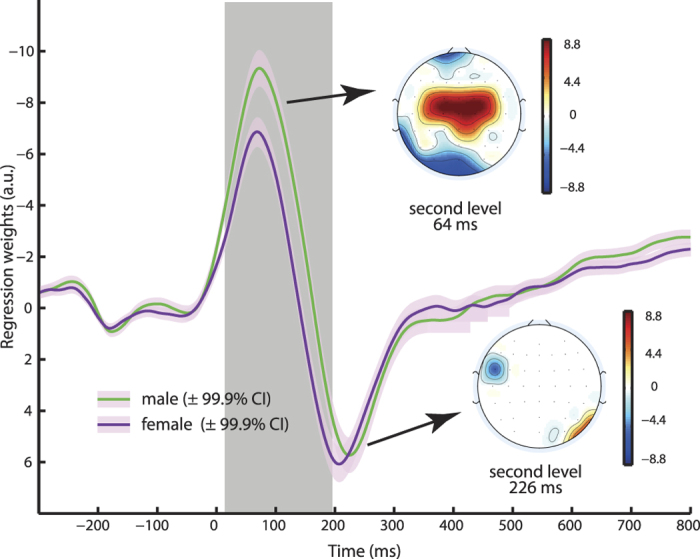
Results of Second Level Regression Analysis for Sex Effects. Displayed are mean regression weights of the error regressor at electrode Cz from the first level analysis for males and females separately. Larger error-related activity was found in male subjects during the time of the ERN and the effect showed a fronto-central scalp distribution (upper topography). Apart from this error regressor effect in the ERN time, no other time points including the Pe (226 ms) showed significant differences (lower topography). The topography plots display second level regression weights and all non-significant (p > 3.3 × 10^−5^) electrodes are masked out in white. The grey shaded area marks the time of significant effects that survived Bonferroni correction. Note that the analysis included factors age and error number as regressors of no interest on the second level, which did not significantly alter activity at both time-points at this electrode.

We then used a second level regression model including each participants’ sex, age, and the number of committed errors as predictors to model first level results of the error regressor. To determine effects, we used the exact time of global maximum effects (Fig. 2B) from a contrast versus no effect of the first level model. We found a significant effect for predictor sex with a peak observed at electrode Cz. Here, at the time of the maximum effect of the error regressor across all subjects (64 ms), men displayed significantly higher error-related brain activity (Fig. 3, robust regression *t*_859_ = 7.14, p < 10^−11^, averaged regression weights for female subjects: −6.9 ± 3.6, males −9.3 ± 4.4, Cohen’s d = 0.60). No sex effect was observed at the peak of the Pe effect (226 ms, *t*_859_ < 0.1, p = 0.98) and additionally participants’ age did not significantly modulate error regressor time-courses (all corrected p > 0.05).

We then included an additional regressor into the model that controlled for each participants’ average RT in order to investigate whether the behavioural difference in RT may explain the differences seen in error-related brain activity. We found that RT itself significantly influenced error regression weights and participants with lower RT showed higher amplitudes (64 ms at electrode Cz robust regression” instead of just “robust regression *t*_859_ = 10.75, p < 10^−24^). While inclusion of RT reduced the sex effect on error-related brain activity, it remained significant (*t*_859_ = 5.26, p = 1.79 × 10^−7^), indicating that male participants showed higher error-related brain activity in the ERN time window over and above also displaying lower RT. See Supplemental Material for an analysis of regular error-related ERPs.

### Gender Prediction based on Multivariate Pattern Classification

**Figure 4:**
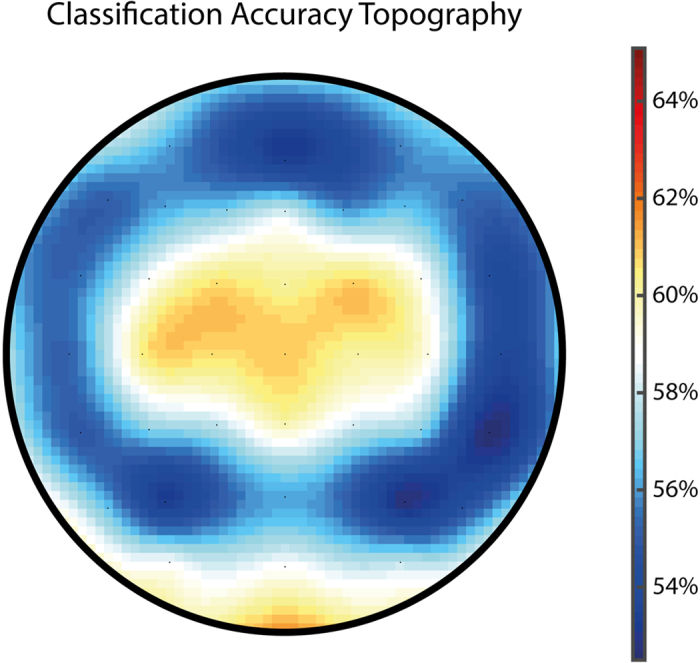
Prediction of Gender by Error-Related Brain Activity. The multivariate classification accuracy based on all sensors was 71.6% based on 500-fold cross-validation. The topography map of accuracies suggests that the main informational content for prediction of a subjects’ gender based on error-related brain activity was located at central electrodes, overlapping closely with the ERN topography.

Given the current debate whether or not a dimorphic distinction between male and female brains is a valid category, we also thought to assess whether or not these statistical differences could be employed to form a categorical distinction. Therefore, we used multivariate pattern analysis of the peak latency error regression weights of the whole scalp to train a support vector machine on the prediction of participants’ genders. Using 500-fold cross-validation of the data split into training (90%) and independent test sets (10%), we found that the brain response to errors was sufficient to predict a subjects’ gender with 71.6% accuracy (chance = 50.0%, permutation test p = 6.67 × 10^−5^). A searchlight analysis of the scalp distribution of this information was in accordance with the well-known ERN topography (Fig. 4).

### Coupling Between ERN and Post-Error Slowing

**Figure 5:**
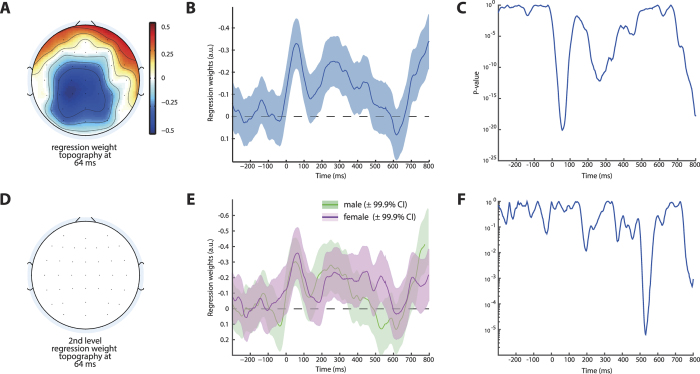
Coupling Between Neural Signals and Consecutive Adaptation. (**A**,**B**) Robust regression coefficients indicate across all subjects that the amplitude of the ERN signal on a given error trial covaries with the following trial’s RT. Thus, higher post-error slowing following error trials is associated with higher ERN amplitudes. This effect is reflected in a negative covariation with a centro-parietal scalp topography (**A**) and minimal p-values coincide with the time of the ERN peak (**C**). When investigated for gender effects, we found that the coupling between ERN and post-error slowing was indifferent between men and women (**E**) and no data point survived correction for multiple comparisons at the peak of the ERN (**D**,**F**). Note that the effect apparent at the end of the displayed time-window (around 700 ms) as well as the effect around 300 ms likely reflect the actual onset of the next response captured by the regressor itself. Scalp topographies are thresholded at p< = 3.3 × 10^−5^, shades represent 99.9% CI.

Next, we investigated possible functional consequences of this differential brain response. We first sought to establish the relationship of single-trial ERN amplitudes to subsequent behavioural adaptation. Therefore, we regressed error-related EEG activity at each data point onto reaction times following error trials including factors of no interest (congruency, response stimulus interval). As expected, we found a negative covariation between EEG amplitudes in the ERN time range displaying a typical scalp topography (Fig. 5A) and consecutive RT (Cz peak 56 ms, b = −0.33, CI = −0.22– −0.44, p = 7.95 × 10^−21^, Fig. 5B,C) strengthening the relationship between ERN and consecutive adaptation in accordance with other studies^20^,^21^. This result confirms that higher, i.e., more negative, ERN amplitudes are associated with higher consecutive RTs. However, we found no gender differences in the strength of this coupling (Fig. 4D,E, robust regression at 56 ms *t*_859_ = −0.50, p = 0.62 uncorrected) suggesting that the degree to which ERN amplitudes influence behavioural adaptation is similar in males and females.

Additionally, in order to clarify similarities between within- and across-subject associations of ERN and PES, we also quantified post-error slowing within subjects (as described above) and regressed it onto error-related brain signals across subjects. However, this analysis revealed no significant association between ERN or Pe related EEG activity and interindividual variance in post-error slowing (all corrected ps > 0.05 at electrode Cz).

### Response Conflict Processing

**Figure 6:**
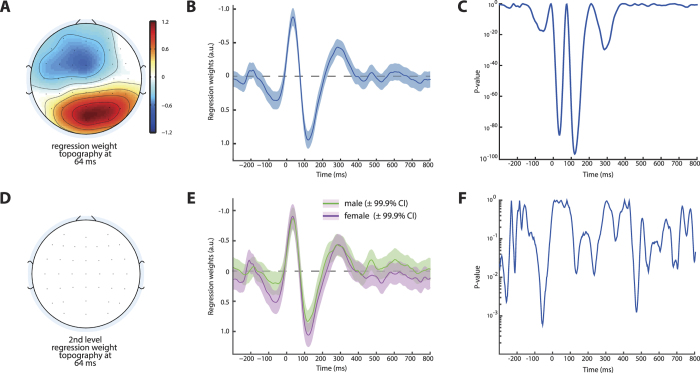
Response Conflict Processing. Displayed are results of a first level regression model on error trial EEG for a regressor that coded for the distance between flanking and target arrows. Larger distances caused significantly (**C**) increased (more negative) ERN amplitudes (**B**) likely due to increased response conflict in this condition^38^, and the effect showed a fronto-central scalp distribution (**A**). However, no difference between male and female participants was found in a second level regression on this factor (**D**–**F**) indicating that differences in conflict processing cannot explain the observed difference in ERN amplitudes between genders.

A possible explanation for the observed gender difference in error-related brain responses could be based on possibly differential response conflict sensitivity or processing between groups, because previous studies found increased ERN amplitudes to be associated with increased response conflict^38^,^39^. Therefore, we compared the degree of error-activity modulation induced by the manipulation of the distance between flanking and target arrows, a parameter found to reflect response conflict as suggested by computational modelling^38^. As expected, we found a strong effect of distance on error-related activity in the early time of the ERN (Fig. 6A–C), which was larger on trials where flankers appeared further away from the target stimulus thus inducing high response conflict (Cz peak at 34 ms, b = −0.88, CI = −0.75– −1.01, p = 8.36 × 10^−86^). However, we did not observe any difference in this measure depending on gender (Fig. 6D,E, p-value at peak 0.85 uncorrected). This suggests that response conflict processing is comparable between both genders.

### Comparison of Variances

Another explanation for the observed gender differences could be that women show more variability in their behaviour^24^, and possibly also in electrophysiological responses – which could corrupt ERP averages and regression results. Therefore, we compared the variance of RTs as well as the within-and across-subject variance in the latencies of error-related brain responses. However, due to RTs generally tending to deviate from normal distribution and a high correlation between mean RTs and SDs across subjects (r = 0.63, p < 10^−96^), we log-transformed RTs prior to analysis. We found a small effect of gender on RT variance (b = 0.19, CI = −0.01–0.40, p = 0.026) indicating slightly higher variances in female subjects. We also compared the difference in variance between correct and post-error trials, obtaining a similar result (b = 0.23, CI = 0.00–0.47, p = 0.013). Furthermore, we included SDs as a separate regressor for the RT analysis across subjects and the effect of gender remained significant (b = 0.29, CI = 0.12–0.47, p = 7.74 × 10^−7^). Thus, the reported gender differences for RT and PES cannot be explained by increased variance in behaviour. Note that log-transformation did not qualitatively change RT results reported above.

For EEG latency measures, we compared the latency of minima in individual trials in the 60 ms surrounding the grand average error-related peak activity. We found no evidence of increased latency variation within female subjects (SD men = 10.5, women = 10.6, b = 0.11, CI = −0.13–0.34, p = 1 corrected). As the same may apply to across group comparisons, we also compared variances of latencies of regression weight minima across male and female participants using Bartlett’s test. This revealed no evidence of a group difference (M men = 67 ± 15 ms, M women = 68 ± 15 ms, p for difference of variance = 0.96, see Supplemental Material for an analysis regarding Pe latency). These findings rule out spurious test statistics induced by confounding differences in within- and across-group variances.

## Discussion

We found evidence for differences in performance monitoring functions between men and women both on a behavioural as well as electrophysiological level. Behaviourally, men were found to respond faster in the task overall whereas no difference in error rate was observed. Additionally, we observed an overall negative association between RT and error rate, indicating that subjects who responded faster also made more errors. Thus, independent of this general effect, men performed the task more efficiently. This finding fits well with several other studies that found faster responding in male subjects employing a Flanker task in adults^26^,^32^ and children^11^ and additionally with behavioural studies employing a variety of RT paradigms^29^,^40^.

A possible explanation for this finding would be the ‘extreme male brain’ hypothesis^41^, which implies that women are more easily distracted by semantic as well as social cues compared to men due to more efficient, autonomic processing of these stimuli. Support for this idea comes from results of an attentional cueing paradigm using arrow stimuli^30^ and another study that employed a Flanker Task^31^. We find some evidence supporting this idea in that the difference between congruent and incongruent trials’ RTs, and therefore the RT cost of conflicting stimuli, was slightly larger in female subjects. However, this effect was much smaller compared to the overall difference in RTs and, thus, this finding cannot be reduced to an explanation based on distractibility alone. Furthermore, the overall RT difference was present when the analysis was restricted to only compatible trials. Thus, in part women appear slightly more distractible by conflicting stimuli, but in addition to this, males respond more rapidly in a Flanker Task.

Furthermore, we find pronounced gender differences in post-error slowing over and above the general observation that subjects responding slower also display higher post-error slowing. Post-error slowing is usually interpreted as an adaptive mechanism that –under appropriate circumstances– provides time for task specific adjustments^15^. This interpretation receives strong support by the observation that post-error slowing correlated positively with increased accuracy following errors in the current task (Supplemental Material). Another study found increased post-error slowing in women comparing 285 male and 346 female subjects in a Stop-signal task^24^. This was interpreted as increased flexibility in setting speed-accuracy trade-offs in female subjects. However, another account of post-error slowing is that it merely represents an orienting response to infrequent events^42^. We find that female subjects do not show larger increases in accuracy following errors, yet also do not decrease in accuracy as could be expected when assuming higher distractibility^11^. Thus, our data support the view that in general post-error slowing provides means to increase performance, but it may also reflect distraction by an infrequent event. Given these findings, it appears that the increase in distraction drives the observed higher post-error slowing in female subjects, which cancels out the possibly adaptive effect and thus does not lead to higher PIA.

On an electrophysiological level, we found increased error-related brain activity related to the ERN, but not Pe, in male subjects. An earlier study reported increased ERN as well as Pe amplitudes in males using a difference-wave approach^26^. However, such a finding could be explained by general morphological differences, for example a larger ACC – as has been found when comparing males and females^27^. Yet, although the neurogenerators giving rise to the Pe are less well established than those of the ERN^43^, the dissociation observed in ERN and Pe time windows here speaks against such an unspecific effect. Importantly, the regression approach both on a first and second level increases specificity of the effect to actual errors. Firstly, we analyse the effect of erroneous responses over and above that of RT and other noise factors, such as the current trial’s congruency. Furthermore, on the second level we find that the sex effect exists over and above possible effects of age or error rate and even when individual RT is included as a separate regressor – which we found to be itself influenced by sex across subjects. Finally, one could be concerned that increased variance in female subjects^24^, which could be quite plausible given that most studies did not control for the state of the participants’ menstrual cycles, may corrupt these electrophysiological measurements. However, the striking absence of such latency variability of EEG measures excludes this explanation. Thus, we report evidence in a large sample of subjects indicating that male subjects display increased error-related brain responses compared to females, while this does not extend to the time of the Pe. Furthermore, this difference translates into the possibility to distinguish male and female brains categorically with an accuracy of ~72% only based on their electrophysiological response to errors. We suggest that such analyses are an important contribution regarding the question of the validity of a male/female brain dimorphism^2^,^3^.

An association between ERN and trait anxiety has been well documented^44^,^45^ and additionally this association has been shown to be modulated by gender in a recent meta analysis^46^. This study found that anxiety scores positively covary with ERN size in females, but not males. Additionally, some studies report higher self reported anxiety in females^26^,^47^. However, it is unlikely that confounding differences in anxiety could possibly explain our data, as one would expect the opposite finding based on speculative differences in our study sample if females are more anxious and higher anxiety covaries with increased error-related brain activity. Yet, one limitation of the current study is that we did not control for possible differences in anxiety or depression. However, one previous study reported gender differences in ERN amplitudes over the effect of anxiety^26^ and the current study appears sufficiently large to render coincidental group differences in a rather homogeneous healthy population unlikely.

This relates closely to another possible explanation of the finding of increased brain responses to errors in male participants, which could be latent, undiagnosed pathology with different prevalences in men and women. However, it is especially surprising that our result is opposite to gender differences in disorder prevalence associated with error-related brain responses. For example, impulse control disorders and substance abuse are associated with reduced ERN amplitudes and more prevalent in male subjects^7^,^48^. Vice versa, anxiety disorders and depression are associated with increased ERN amplitudes^49^ and more common in females^9^. Therefore, it is highly unlikely that the finding of an increased error-related brain response in males is caused by latent, underlying pathology as this would likely shift results in the opposite direction. Yet, especially the highly desirable discovery of endophenotypes, predictive of disorder onset as demanded by the RDoC, needs to carefully control for such gender differences and future studies need to carefully consider possible interactive effects between gender and neural measures^1^.

However, it still remains an open question what these neural differences translate into and how they may arise. When we investigated the association between ERN and consecutive RT changes, we found convincing evidence showing that single-trial ERN amplitudes covary with the degree of consecutive slowing. This is in accordance with several other studies^20^,^21^ and given the temporal relation of both phenomena, suggestive of a causal relationship. Although this within subject association offers the most stringent correlational test of brain-behaviour interactions^50^, analyses of absolute amplitudes across subjects do not reflect the same effect. Neither here, nor in most other studies of across subject association between error-related brain activity, was it found that ERN (or Pe) amplitudes correlate with PES^51^. Our data suggest that despite a shift in overall error-related activity seen in male subjects, and higher PES in females, the association between error-related EEG activity and post-error slowing is equally strong in both sexes. One possible explanation of this constellation could be that differences in response conflict processing drive the observed increase in error-related EEG dynamics in males. However, despite clear evidence of effectivity of the employed manipulation of stimulus distance, which varies the degree of response conflict, we found no evidence of differential gender effects here. On a side note, the observed topographical and temporal difference between ERN modulation by response conflict (Fig. 6A) and the covariation between ERN and PES (Fig. 5A), hints at different sub-processes reflected in EEG activity in the time of the ERN, which is usually considered as a single entity. In sum, despite solid evidence for male subjects displaying increased error-related brain activity, the functional relevance of this finding remains an open question, although we can rule out several potential explanations.

This specific gender effect on the ERN is certainly important for any study that compares error-related brain activity across samples of subjects (e.g., clinical populations). Additionally, our results demonstrate that future studies need to disentangle directly RT mediated effects on ERN amplitudes both within and across subjects. It is an open question whether these differences are genetically determined or emerge through environmental influences. For ERN and RT, heritability estimates range between 40 and 60%^52^,^53^, but future studies should more precisely investigate the influence of specific genes and their interaction with participants’ sex on these factors^4^. Future work should also focus on potential sex differences in cortical microcircuitry that might explain stronger summed-up EEG signals even in the absence of functional effects on behaviour.

## Methods

### Participants

895 healthy human subjects (aged between 18 to 40 years; central European ancestry) that gave written informed consent to participation were included in the study. Exclusion criteria were: history of psychiatric and/or neurological disease; regular use of medicine; relevant history of drug abuse (relevant: consumption within the last month or more than occasional consumption (more than 5 times in lifetime, without cannabis); regular (more than one per month) consumption of cannabis; alcohol intake at day of study; caffeine consumption less than three hours before experiment). All study procedures were approved by the ethics committees of the University of Nijmegen (ECG04032011), where 388 datasets were collected, and the University of Leipzig (285-09-141209), where all other datasets were collected, and all procedures were carried out in accordance with the approved study protocol.

Grubbs’ test for outlier detection^54^ with a one sided alpha of 0.01 was applied to exclude subjects with a too high proportion of missed trials (>16%; found in n = 9 subjects) in the Flanker Task. The same test was used to exclude subjects with a too high proportion of trials with more than one response (n = 12) in order to exclude subjects that did not follow task instructions well or were not focused on the task. Datasets with broken central channels and where ICA did not converge were furthermore excluded from EEG analysis (11 subjects). Thus, the final sample consists of 863 subjects for EEG and 874 (438 female, 436 male) subjects for behavioural analyses. The mean age of the sample was 24.2 (range: 18–40) years and male subjects were slightly older (25.5 ± 3.6 (SD) years) compared to female subjects (22.9 ± 3.7 years).

### Task

A speeded arrow-version of the Eriksen Flanker task known to induce a sufficient number of erroneous responses as well as post-error slowing^15^,^38^ was employed. Participants had to respond according to the direction (left or right) of a centrally presented arrow (target) that appeared on screen for 33 ms and ignore 4 flanking arrows that appeared above and below the target 83 ms earlier. The size of all arrows was 1.9° × 1.3° of visual angle. The task consisted of 1080 trials and on half of these the direction between flankers and target was identical (congruent trials) whereas they pointed in the opposite direction on the other half of (incongruent) trials. The distance between flanker and target stimuli was modulated in two conditions between far (flanker-target distance: 6.5° and 4°) and close (3.5° and 1.75°). The time between response and onset of the next trial (response-stimulus interval, RSI) was also modulated in two conditions (250 and 700 ms). Congruency and flanker-target distance and their respective transitions were counterbalanced in pseudorandom order. Fifty per cent of congruent trials were preceded by a short and 50% by a long RSI (same for incongruent trials), 50% of far trials were preceded by short, 50% by long RSI (same for close trials). We then excluded all trials with RTs < 100 ms or >1000 ms or in which subjects responded more than once from behavioural and EEG analyses. For EEG analyses, on average 142 error trials were included per subject and this did not differ between female (mean 141, range 24–280) and male (mean 144, range 37–319, t-test for difference *t*_861_ = 1.09, p = 0.27).

### Behavioural Analyses of Sex Effects

For the analysis of RTs with regard to sex, we first compared RTs on all correct trials that neither followed nor preceded errors. Post-error slowing was calculated by subtracting mean RTs of all correct trials preceded and followed by correct trials from mean RTs on correct post-error trials. Results were then submitted to multiple linear regression analysis across subjects with separate predictors accounting for participants’ age, the total number of committed errors, as well as the general RT for the analysis of PES to assess if the effect existed over and above possible differences in RT on all trials.

### EEG Recording

Elastic EEG caps (Easycap, Brain Products) with 60 Ag/AgCl sintered electrodes were mounted in the extended 10–20 system with impedances kept below 5 kΩ. Data were recorded continuously at a 500 Hz sampling rate with BrainAmp MR plus amplifiers (Brain Products) and analysed offline using EEGLAB 13^55^ as well as custom code written in MATLAB 2015a (MathWorks). Electrodes at the left and right outer canthus and above and below the left eye captured eye movements. The ground electrode was positioned at the sternum, data were online referenced to A1 and offline re-referenced to common average. The task was performed while participants were seated in a dimly lit and acoustically shielded room.

### EEG Data Analysis

EEG data was filtered with a 0.5 Hz high- and 42 Hz low-pass filter. Epochs from 1.5 s before until 2 s after target onset were then extracted. Epochs containing deviations >4 SD of the mean probability distribution of all error and correct trials in a shorter time window surrounding the stimulus (−300 to 1000 ms) were automatically rejected, yet no more of 5% of the trials in each condition were excluded and otherwise the rejection threshold was increased.

Data was then demeaned and subjected to adaptive mixture independent component analysis^56^. Representative independent component topographies reflecting blink, horizontal eye movement and bipolar eye artefacts were chosen and used as templates for CORRMAP – a semi-automatic component identification algorithm^57^. All individual datasets were thereafter visually inspected by at least one human researcher acquainted with EEG methodology who selected additional independent components reflecting other, less homogenous noise sources for removal. Following baseline correction (−250 until −150 ms locked to response onset), the data was then used for multiple robust single-trial regression analyses^20^,^37^.

### Regression Models for EEG Data

For EEG analyses, we employed multiple robust regression first within and then across subjects. The first level EEG model included, apart from a regressor coding the current trials accuracy, the following regressors to account for factors of no interest: the current trial’s congruency (congruent/incongruent), distance between target and flankers (close/far), log scaled RT, response hand (left/right), RSI (short/long), as well as the following trial’s RSI. From this model, we used the regression weights for the error regressor, scaled by its respective SE, for a second level regression analysis including factors sex, age, and number of errors. For analysis of post-error slowing with regard to EEG amplitudes, we regressed the EEG amplitude together with the following trials’ congruency, RSI, and distance between flanker and target stimuli onto the actual RT in order to control for these effects. Again, resulting robust coefficients were then submitted to second level analysis. For first level regression analyses, data was smoothed with a running average of 10 ms surrounding each data point. No further smoothing was applied for second level analyses.

### Support Vector Machine

We trained a support vector machine to classify a subjects’ gender based on the multivariate brain response to errors, i.e., the regression weights for the error regressor of the first level analysis, measured at peak latency. We used the Matlab functions fitcsvm/predict using a linear kernel on a feature vector consisting of the regression weights of all participants at 59 electrodes (excluding eye-channels) and 500-fold cross-validation using 90% of the data as training and the left 10% as independent test sets (i.e., 43 random participants). Accuracy was calculated as the percentage of overlap between predicted labels and the ground truth (i.e., a subject’s true gender). Random subsampling (to 429 participants per class) was used to ensure that both classes consisted of the same number of entries and chance classification is thus exactly 50.0%. Data was scaled from −1 to 1 across all channels and participants to accelerate computation. Resulting accuracy was statistically tested using a permutation test with random class ascriptions and 15.000 iterations. To determine the informational content of each electrode, we adopted a “searchlight” approach: we calculated the average accuracy (using 50-fold cross-validation) for each electrode alone (i.e., a univariate classification), every lateralized electrode together with its contralaterally located electrode, and each electrode clustered with the 7 nearest neighbouring electrodes. This resulted in an average accuracy per electrode, which is plotted as a scalp topography in Fig. 4.

## Additional Information

**How to cite this article**: Fischer, A. G. *et al*. Gender Influences on Brain Responses to Errors and Post-Error Adjustments. *Sci. Rep.* **6**, 24435; doi: 10.1038/srep24435 (2016).

## Supplementary Material

Supplementary Information
