# Supplementary material for: Gender Influences on Brain Responses to Errors and Post-Error Adjustments
Source: Sci Rep. 2016 Apr 14;6:24435. doi: 10.1038/srep24435 (PMC4831004; doi:10.1038/srep24435)
Supplement: Supplementary Information [file srep24435-s1.pdf]

Supplemental Material for

Gender Influences on Brain Responses to Errors and Post-Error Adjustments

Adrian G. Fischer, Claudia Danielmeier, Arno Villringer, Tilmann A. Klein, Markus Ullsperger

General Task Effects. The average mean RT across all subjects was 371 ± 34 ms (SD). Subjects were slower on incongruent trials (Δ +38 ± 13 ms, p = 0 within machine precision) which also induced most of the errors (error rate incongruent 17.0%, congruent 3.2%). RTs following errors were significantly increased compared to post-correct trials confirming the presence of post-error slowing in the task (Δ +54 ± 35 ms, $p = 2.11 \times 10^{-233}$). Accuracy was increased following errors (relative Δ +38 ± 8%, $p = 2.10 \times 10^{-235}$) and the increase in accuracy correlated positively with the amount of post-error slowing (r = 0.18, $p = 1.34 \times 10^{-7}$), indicating that slowing following errors provided more time for task specific adjustments.

Additional Post-Error Slowing Effects. Another way of calculating post-error slowing is to subtract RTs immediately preceding errors from the RT following errors which accounts for the possibility of local fluctuations in attention. Results for the factor sex were not qualitatively different and the sex effect remained significant (b = 0.37, CI = 0.15 – 0.60, $p = 5.27 \times 10^{-7}$).

ERP Latency Effects. Females did show a very tiny, yet significant, effect of earlier ERN (men = 63 ms, women = 61 ms, b = -0.53, CI = -0.76 – -0.30, $p = 8.83 \times 10^{-13}$) and especially Pe amplitudes (men = 231 ms, women = 221 ms, b = -0.57, CI = -0.79 – -0.34, $p = 8.29 \times 10^{-15}$) when measured as individual trial minima on erroneous trials. However, latencies of regression weight minima in the ERN time window across subjects, the primary measure we compare in the analysis, where indifferent between male and female participants (mean men = 67 ± 15 ms, mean women = 68 ± 15 ms, p = 0.22 for difference in means). For Pe latencies, yet, we found an earlier peak for women (men = 240 ms, women = 224 ms, b = -0.47, CI = -0.70 – -0.24, $p = 3.18 \times 10^{-10}$) as well as increased latency variation in female subjects (SD men = 25 ms, women = 31 ms, T = 15.7, $p = 7.27 \times 10^{-5}$). As such a pattern might confound comparisons of regression weights at single points in time, we also report a comparison of individual regression weight minima in the Pe time window (226±60 ms). However, this revealed no hint at gender differences in Pe amplitudes (b = -0.03, CI = -0.27 – 0.21, p = 0.68 uncorrected).

Study Centre. All procedures in both study centres (University of Nijmegen, Netherlands and Max Planck Institute for Human Cognitive and Brain Sciences, Leipzig, Germany) were carried out with an identical EEG recording setup and by the same technical staff. However, both study populations differed in the distribution of factor gender: the Dutch sample comprised ~80% female, whereas the German sample only 30% female subjects, correlation *gender* and *centre* r = 0.48). Thus, we repeated all second-level analyses where we found significant sex effects with inclusion of a separate factor *centre* coding the place of data collection. Although one would expect a certain reduction in effect size due to autocorrelation, all reported effects remained significant.
The difference in average correct trials´ RT was less robust here (b = 0.26, 99.9% CI = 0.03 – 0.50, p = 0.003) and the study centre revealed a significant effect on correct RT such that participants measured in the Dutch centre displayed higher RTs (ΔRT 19 ms, b = -0.43, CI = -0.68 – -0.17, $p = 5.77 \times 10^{-7}$). There was no effect of the study centre on post-error slowing (p = 1 following correction) and the sex effect remained

unchanged (b = 0.44, CI = 0.20 – 0.69, $p = 6.11 \times 10^{-8}$). Second-level robust regression results at the peak of the ERN time again were very similar to the initial analysis for the comparison between both genders (robust regression $t_{859} = 6.09$, $p = 1.68 \times 10^{-9}$) and regressor centre did not show a significant effect on the ERN (p = 0.16) nor Pe time window (p = 0.80).

Therefore, although an effect of the place of measurement on the overall RT was observed, and this somewhat reduced the gender difference, all main findings of the study remained significant over and above the slightly confounding difference between the two data collection centres. It should be noted additionally that the prior likelihood and assumption of cognitive differences between a rather homogenous mainly Dutch and German European population is low, which is why we report this finding as a supplementary analysis.

Complete First Level Regression Model. The first level EEG model included, apart from a regressor coding the current trials accuracy, the following regressors to account for factors of no interest: the current trial´s distance between target and flankers (close / far), congruency (congruent / incongruent), log scaled RT, response hand (left / right), RSI (short / long), as well as the following trial´s RSI (Figure S1).

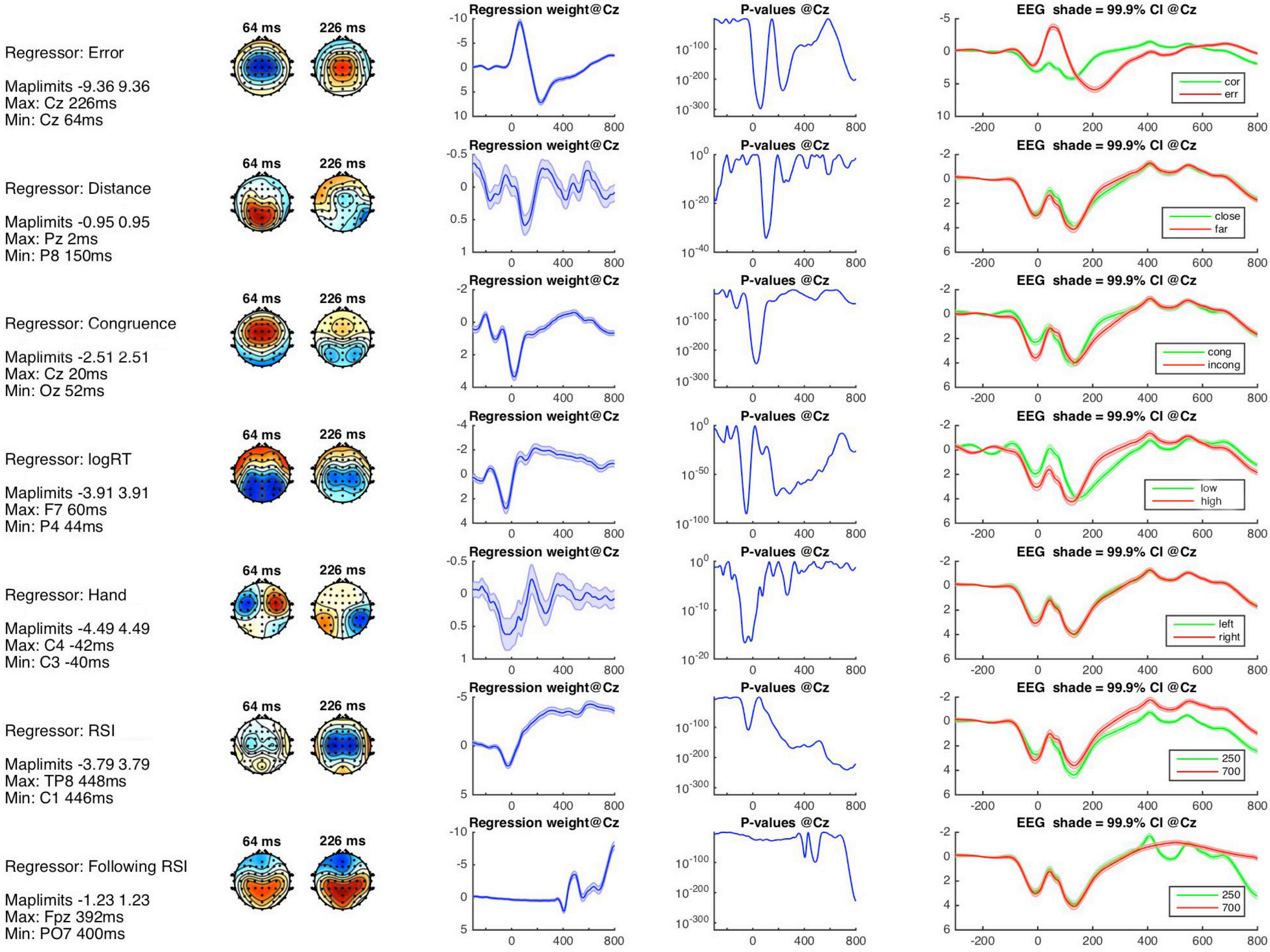

Figure S1 | Results for the Complete First Level Regression Analysis. Columns reflect (from left to right): Regressor name, map limits of topography plots (a.u.) and time and electrode of maximum and minimum activity across the scalp; topographies at ERN and Pe peak time; regression weight time course at electrode Cz (shades = 99.9% CI); associate point-wise p-values for a contrast against no effect; grand-average EEG activity for comparison split by each regressor.

Analysis of Late Pe Activity. The Pe can be subdivided into an early, ERN-like peak, and a later, more parietally peaking part {OConnell:2007jp, Arbel:2009jh}. In order to investigate possible differential effects of subjects´ gender on this later Pe, we also report first- and second-level effects for this factor here.

We found the maximal effect of the error regressor at 320 ms following response onset at electrode Pz, where a centro-parietal scalp distribution was evident (Figure S2A) that showed a longer lasting difference between correct and erroneous responses up until ~600 ms after the response (right plot Figure S2A). We then submitted peak regression weights (at 320 ms and electrode Pz) to second-level analysis for factor *gender* (again including the regressors *age* and *error number* as regressors of no interest). This, as for the early Pe, revealed no influence of a participants´ gender on late Pe amplitudes (robust regression t_{859} = 1.45, $p > 0.14$).

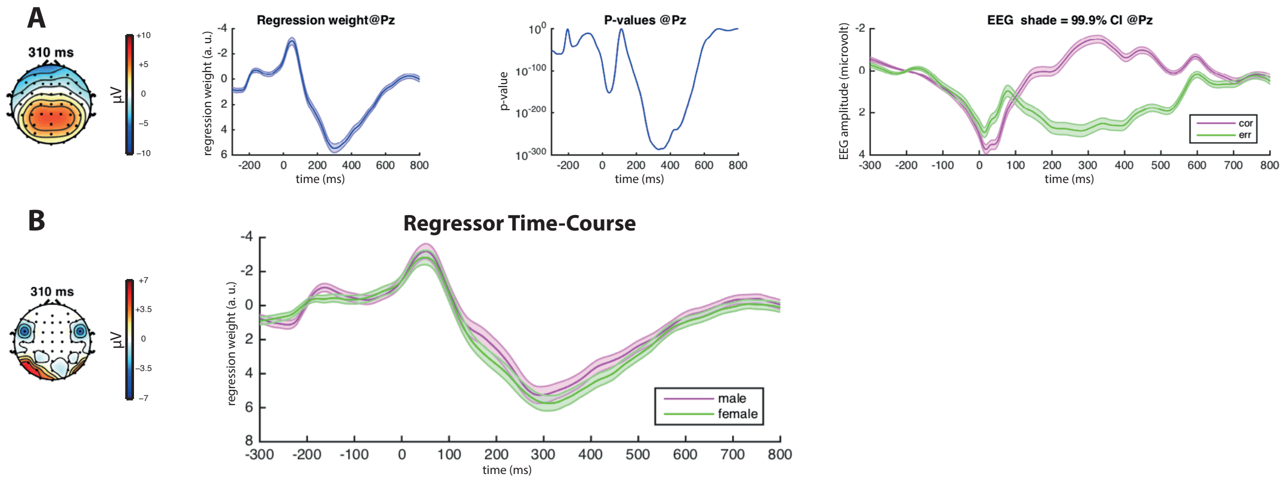

Figure S2 | Analysis of Gender Effects on the Late Pe. The late Pe showed an expected centro-parietal scalp distribution and a peak at 310 ms at electrode Pz (A, left topography). Regression weight time-courses, associated p-values and EEG activity (three right plots in A) indicate a long lasting significant difference between correct and erroneous responses. However, second-level robust regression did not confirm an influence of factor gender at centro-parietal electrodes (topography, non-significant time-points masked in white) nor over the time course of the regressors for males and females (shades = 99.9% CI).

ERP Analysis. For completeness, we also report results of a conventional ERP analysis. Therefore, we calculated grand-average waveforms for all errors on incongruent trials separately for male and female subjects. ERN amplitude was largest at electrode FCz (Figure S3) and we measured the ERN as the mean amplitude from 50 to 70 ms following the response. On error trials this revealed significantly larger amplitudes in male (-5.37 ± 0.18 µV ± SE) compared to female participants (-4.70 ± 0.16 µV, t_{861} = 2.71, p = 0.0059). For CRN amplitudes, no such effect was evident (t_{861} < 1, p = 0.96) as male (1.43 ± 0.12 µV) and female (1.42 ± 0.12 µV) participants displayed similar amplitudes. This suggests that the increased brain response to errors of males we report in the main regression analysis is indeed caused by differences on erroneous as opposed to correct responses.

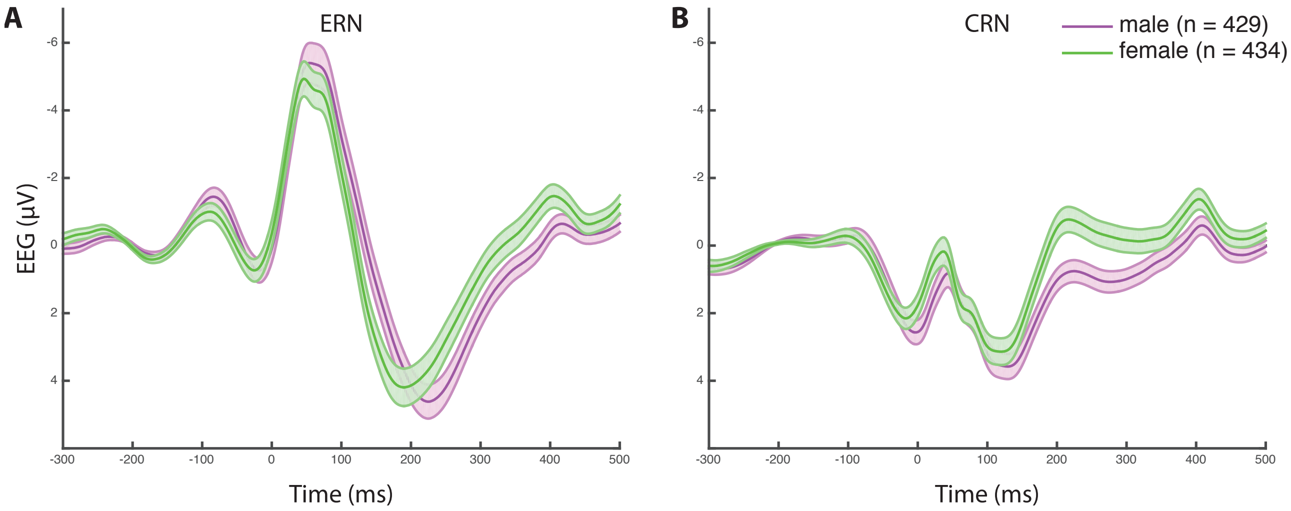

Figure S3 | ERPs for Error and Correct Responses. Plotted are ERPs locked to response onset on error (A) and correct (B) trials at electrode FCz for male (pink) and female (green) subjects. Males had increased ERN amplitudes. Shaded Area = 99.9% CI.
